# Strain-dependent assessment of dough’s polymer structure and functionality during the baking process

**DOI:** 10.1371/journal.pone.0282670

**Published:** 2023-03-07

**Authors:** Thekla Alpers, Thomas Becker, Mario Jekle

**Affiliations:** 1 Research Group Cereal Technology and Process Engineering, Chair of Brewing and Beverage Technology, Technical University of Munich, Freising, Germany; 2 Department of Plant-Based Foods, Institute of Food Science and Biotechnology, University of Hohenheim, Stuttgart, Germany; University of Sharjah, UNITED ARAB EMIRATES

## Abstract

During the baking process, the functionality of the heterogeneous dough matrix changes as the composing polymers experience conformational transition processes. The thermally induced structural changes affect the involvement and functionality of the polymers in the dough matrix. With the main hypothesis being that different types and magnitudes of strain exerted during the measurement would provide information on different structural levels and interactions, SAOS rheology in multiwave mode and large deformation extensional rheometry were applied to two microstructurally different systems. The functionality of the two systems, a highly connected standard wheat dough (φ ≈ 1.1) and an aerated, yeasted wheat dough (φ ≈ 2.3), depicting limited connectivity and strength of interactions, was accessed under different deformations and types of strains. Applying SAOS rheology, starch functionality prevailed on the behavior of the dough matrix. In contrast, gluten functionality prevailed the large deformation behavior. Using an inline fermentation and baking LSF technique, the heat-induced gluten polymerization was shown to increase strain hardening behavior above 70°C. In the aerated system, the strain hardening effect became already evident under small deformation testing, as the expansion of gas cells caused a pre-expansion of the gluten strands. The expanded dough matrix of yeasted dough was further shown to be substantially subjected to degradation once the network reached beyond its maximal gas holding capacity. Using this approach, the combined impact of yeast fermentation and thermal treatment on the strain hardening behavior of wheat dough was revealed for the first time by LSF. Furthermore, the rheological properties were successfully linked to oven rise behavior: a decreasing connectivity combined with the initiation of strain hardening by fast extension processes occurring in the yeasted dough matrix during the final baking phase was linked to limited oven rise functionality prematurely around 60°C.

## 1 Introduction

The functionality of wheat dough is defined by the interactions of starch and protein, the main polymers constituting the dough matrix. Hydration and mechanical energy input lead to the formation of a network structure with viscoelastic behavior. The complex rheological behavior changes during the thermal treatment during baking. The dough-to-crumb-transition is initiated by conformational changes of the consisting matrix polymers. This unique behavior has been used in bread making for centuries. In particular, the viscoelastic properties of the non-Newtonian fluid crucially impact the behavior of dough during shaping, proofing and baking. The properties of the dough matrix are enabled by the interactions of the dough’s biopolymers on different structural scales. The matrix consists of suspended starch granules, which are located in a 3-dimensional gluten network together with minor dough components such as lipids, arabinoxylans and enzymes [1]. Therefore, decisive interactions appear in between starch granules, between starch granules and proteins, and between the supramolecular protein assemblies [2–4]. Depending on the deformation applied to the dough system, relevant interactions respond in a strain-dependent manner. In this way, primarily short- and long-range interactions are distinguished. Short-range interactions (mainly starch-starch interactions) respond at low strains, whereas increasing strains have to be applied to cause a response of gluten-starch and gluten-gluten interactions [2]. Some authors further highlighted the importance of the type of strain applied, as the functionality of gluten is mainly exposed upon extensional deformation [5–8]. Accordingly, extensional deformation has been used to quantify e.g. strain hardening behavior of dough [9–11]. Translating this strain-dependent behavior to the production process, kneading, shaping, proofing and oven rise processes may be considered large deformation processes, which might primarily cause the response of long-range interactions.

Besides the diverse interactions on different structural scales within the dough phase, thermal treatment during the baking process initiates an even more complex behavior by initiating conformational transition processes of the dough’s polymers. Therefore, quantification of the changing material properties by rheological measurements upon thermal treatment is of special importance. Small amplitude oscillatory shear rheology has been commonly used to quantify those rheological changes during the baking process using temperature sweeps. As is the case during kneading, shaping, proofing and oven rise, the viscoelastic dough system is also subjected to large deformation. Hence, large deformation experiments can also provide (and presumably better) relevant information. In shear rheology, this behavior can be accessed using LAOS rheology [12].

Recently, extensional techniques have gained increasing popularity in cereal technology as the dough matrix mainly undergoes biaxial extension during the bread making process. This deformation is responded by specific functionalities, originating from the polymerized character of the gluten proteins. Here, strain hardening behavior is important, which is widely known in polymer science and defined as a phenomenon that the stress required to deform a material increases more than proportional to the strain at a constant strain rate [13]. Therefore, elongational rheology can provide further information on the behavior of dough during process-relevant deformation types [11]. In this way, extensional techniques, such as lubricated squeezing flow, capillary breakup elongational rheometry, filament stretching rheometry, sentmanat extension rheometry or hyperbolic contraction flow can principally be applied [9,11,14–17]. Especially in order to access gluten (or zein) functionality, these techniques are state-of-the-art [7,10,17–19].

So far, little research has been conducted on the impact of thermal treatment on the elongational rheological behavior of wheat dough. Recently, Vanin et al. (2018) successfully conducted LSF measurements to trace the changing rheological behavior of non-yeasted wheat bread dough during thermal treatment and observed marked differences in the shear rheological behavior of dough during heating [17]. So far, research has focused on the behavior of highly connected polymeric wheat dough systems under elongational deformation upon thermal treatment. Conversely, the integrity of the wheat dough network cannot be granted in practice, as yeast metabolites, generated during the fermentation process, affect the functionality of the gluten network along the bread making process [10,20]. The impact of the degradation of the well branched structure on wheat dough functionality has not been quantified yet and will be the focus of this work.

Hence, this paper aims to elucidate the link between dough structure and oven rise behavior by shedding light on the polymers’ functionality in relation to the changes in the dough’s molecular and microstructure. Therefore, two dough systems will be characterized using different shear and extensional rheological methodologies. Both systems will be studied with different rheological methods to access their structural and functional changes during the dough-to-crumb-transition. It is hypothesized that different types and magnitudes of strain exerted during the measurement will provide structural information on different scales. The dough systems used in this work are standard non-yeasted wheat dough and yeasted wheat dough. In comparison to the standard wheat dough system, the microstructure of yeasted dough has been shown to be less branched, as the microstructure of yeasted dough is markedly affected by the degrading effects of yeast fermentation [10,21]. Beside this, secondary yeast metabolites were not shown to affect the network structure or functionality significantly [10]. Therefore, yeast represents an optimal tool to implement structural changes, while the functionality of the dough matrix polymers itself remains constant. However, concerning the drawbacks, the measurement of yeasted doughs has been shown to cause major difficulties. Due to the expansion caused by the CO_2_ production and dissolution from the liquid dough phase, the dough structure becomes very sensitive to external forces and a sample transfer to the measuring device after fermentation is likely to cause structural damage. Therefore, it would be desirable to ferment the samples in the measurement device itself (which will subsequently be referred to as inline fermentation) to overcome these disadvantages. In inline fermentation, uniaxial expansion of the sample must be enabled to avoid overfilling effects. In extensional rheology, a suitable technique for inline fermentation, heating and rheometry using lubricated squeezing flow was formerly suggested by Alpers et al. (2021) [21]. Regarding shear rheology, inline fermentation is even more desirable, as sample transfer—and especially sample loading—can cause a degassing of the fermented dough sample. During inline fermentation and baking, normal force driven measurement gap adjustment allows the progressive expansion without erroneous sample compression or loss of sample contact. Furthermore, the implementation of multiwave oscillatory shear rheological techniques favor the measurement of yeasted dough samples. Applying multiwave rheology, more information can be extracted out of a reduced measurement time [22,23]. This is beneficial for accessing the time-dependent functionality of dough during fermentation and temperature-dependent behavior during the baking process. In case of yeasted dough, multiwave rheology is further advantageous due to the short measurement times at the same information content. As normal force adjustment should not be active during shear rheological measurements, a short measurement time comes together with lower downtimes for the normal force adjustment, hence avoiding the biaxial sample expansion. Overall, the knowledge gained on strain-dependent dough functionality in different matrices during the baking process will be linked to the dependency of oven rise behavior on the structure and functionality of the polymeric dough system.

## 2 Materials and methods

### 2.1 Dough preparation

The experiments were performed using a German commercial wheat flour Type 550. Standard flour characterization resulted in a quantified moisture content of 13.30 ± 0.17 g moisture per 100 g flour (AACCi 44–01, n = 3, $\bar{X}$ ± STD), a protein content of 11.26 ± 0.10 g per 100 g dry flour (AACCi 46–16, N × 5.7, n = 3, $\bar{X}$ ± STD), 0.55 ± 0.05 g ash per 100 g dry flour (ICC 104/1, n = 3, $\bar{X}$ ± STD) and 30.5 ± 0.1 g wet gluten per 100 g flour (AACCi 38-12A, n = 3, $\bar{X}$ ± STD). The kneading was performed in a 50 g scale Z-kneader at 63 rpm using 49.60 g flour, 3 g white sugar/100 g flour (EC category II quality, Bäko, Nürnberg, Germany) and 29.25 ml demineralized water. The kneading procedure was adjusted to the optimum dough development protocol which was previously determined using a Z-kneader doughLAB (Perten Instruments AB, Hägersten, Sewden) following AACCi 54–70.01. Sugar was added to provide sufficient fermentable sugars for the yeast fermentation. Yeasted doughs were prepared using fresh compressed yeast (*Saccharomyces cerevisiae*, F.X. Wieninger GmbH, Passau, Germany) at a level of 1 g yeast/100 g flour.

### 2.2 Elongation properties by lubricated squeezing flow

The sample preparation was performed similar to [21] and will be described in the following section. After the kneading process, the dough samples were sheeted to the desired sample height and cut using a cylindrical cutter (45 mm diameter). Standard non-yeasted wheat dough was sheeted to a final height of 20 mm, whereas in yeasted wheat dough the final height was decreased to 10 mm to compromise for volume expansion during fermentation and baking. Pre-tests confirmed comparable heights of the yeasted and non-yeasted dough samples prior to the compression step using this protocol. Furthermore, no significant difference (α = 0.05) has been shown between η_b_($\dot{\varepsilon }$ = 0.1) derived from wheat dough samples of 20, 30 and 40 mm height, proofing the validity of the used approach. The cylindrical samples were then lubricated using paraffin oil (viscous, Merck KGaA, Darmstadt, Germany) and afterwards immediately transferred to a self-constructed rig for a texture analyzer (TA.XT.Plus, Stable Micro Systems Ltd, Godalming, United Kingdom) equipped with a 50 kg load cell. The setup was developed based on the procedure described by Chatraei et al. (1981) [14]. The original setup was refined by integrating a heating unit to allow in-line fermentation and heat treatment of the dough samples. This approach was used to prevent any external stress of the fermented dough samples, which could lead to a degassing and the collapsing of the structure. Therefore, the setup consisted of two lubricated metal plates of 45 mm diameter, which were tempered using electric heating resistors (aluminum housed fixed power wirewound resistor, 1 kΩ, 25 W, ATE Electronics, Giaveno, Italy) connected to a PID temperature controller (SYL-2342P, Auber Instruments, Alpharetta, GA, USA). The samples were fermented for 60 min at 30°C to allow the release of sufficient gas and secondary yeast metabolites into the dough matrix. During the fermentation and baking step, the samples could expend uniaxially as the upper plate was adjusted in order to keep the normal force at a constant level of 10 g. Uniaxial extension of the dough samples was achieved using a silicone jacket (0.5 mm, Sahltec, Bremen, Germany) and a customized, 3D-printed shell (GreenTec Pro, Extrudr FD3D GmbH, Lauterach, Austria). Using these coatings and applying a paraffin layer, the dehydration of the samples was prevented during this time. After fermenting/resting, the dough samples were heated to the desired temperatures. Initial time-temperature calibration of the heating step was performed in terms of pre-tests using an external 1-wire digital thermometer (DS18B20, Maxim Integrated Products Inc., San Jose, CA, USA) connected to an Arduino microprocessor (Arduino Mega 2560, Arduino AG, Turin, Italy), which tracked the dough’s core temperature. These pre-tests were performed in triplicate, both for yeasted and non-yeasted doughs. In the final setup, the dough samples were heat-treated according to the previously measured heating times to ensure the structural integrity of the samples. After reaching the desired baking temperature (30; 50, 60, 70, 80, 85°C, respectively), the silicon jacket and 3D printed shell were removed to allow a free squeeze flow. The cylindrical samples were compressed to 90% of their initial height by displacing the upper plate. The compression was performed using five different displacement speeds (0.1, 1, 2, 5 and 10 mm/s). The true compression speed was adjusted according to the final sample height of each sample and corrected to reach comparable strain-strain rate combinations. The resulting force-displacement-curves were used to calculate the biaxial strain ε_b_, the biaxial strain rate $\dot{\varepsilon_{b}}$, and the apparent biaxial viscosity η_b_* according to [10]. Further, the strain hardening index was calculated by extracting the stress values for deformations of 0.3, 0.4, 0.5, 0.6, 0.7 0.8, 0.9 and 1.0 for each displacement speed and temperature. The stress read values were plotted against the biaxial strain rate using a double logarithmic scale. The data was fitted using a linear model, which was then used to calculate the stress values for two biaxial strain rates (0.01 and 1.00 s^-1^), exemplarily representing rather slow and fast extension processes within the limits of the method. The obtained stress values were plotted against the deformation on a logarithmic scale. According to Rouille et al. (2005), the slope of the linear model expresses the strain hardening index, defined as the increase in stress with increasing extension at a constant extension rate [9]. All measurements were performed in triplicate and the average values are presented with the standard deviation.

### 2.3 Shear rheological behavior accessed by multiwave rheology

The oscillatory shear rheological measurements were performed using a Modular Compact Rheometer (MCR 502, Anton Paar GmbH, Graz, Austria). The rheometer was equipped with parallel cross-hatched plates (25 mm diameter). The temperature and humidity of the samples were controlled by a CTD 180 HR chamber (Anton Paar GmbH, Graz, Austria) connected to a modular humidity generator (MHG 100, ProUmid GmbH & Co. KG, Ulm, Germany). After kneading, 4 g dough were transferred to the rheometer and centered within the measurement geometries. Subsequently, the sample was compressed to an initial height of 2 mm in case of standard non-yeasted wheat dough and to 1 mm in case of yeasted wheat dough (to compensate for volume extension effects) by setting the measurement gap. After removing excess dough, the cutting surface was covered with paraffin oil (viscous, Merck KgaA, Darmstadt, Germany) to prevent dehydration. The samples were then allowed to rest/ferment for 60 min. Applying normal force controlled gap adjustment (F_N_ = 0.01 N), a uniaxial extension of the dough samples was achieved during the fermentation step. Using this gap adjustment, it was possible to maintain the sample diameter equal to the diameter of the plate-plate geometry at any time. This is normally achieved by trimming the sample to a cylindrical shape prior the measurement so it should not be harmed during the subsequent equilibration or measurement steps. The highly sensitive normal force control enabled a free uniaxial expansion of the sample during a 60 min fermentation step and the consecutive heating process. Fermentation was performed at 30°C and 80% relative humidity. Subsequently, a temperature sweep was performed from 30 – 95°C at a heating rate of 4.5°C/min. During the heating steps, normal force control was activated (F_N_ = 0.01 N), whereas the gap was kept constant during the measurement intervals. Measurements were conducted every 5°C at a frequency of 1 Hz and a deformation of 0.05% at a fixed measurement time of 25 s. The deformation was applied as multiwave strain according to Vidal et al. (2022) [23], enabling the simultaneous measurement of multiple frequencies within a single measurement point. Besides the fundamental frequency of 1 Hz, harmonics being the 2^nd^, 3^rd^, 5^th^, 7^th^, 8^th^ and 10^th^ multiple of the fundamental frequency, were performed simultaneously. The resulting amplitude totaled 0.082%, which was still in line with the determined LVE region of yeasted and non-yeasted dough. The Fourier transformation of the superimposed resulting torque/stress functions was automatically evaluated by RheoCompass (Version 1.25, Anton Paar GmbH, Graz, Austria). The obtained values for complex module data (|G*|) were then fitted to the power law equation (c.f. Eq (1)).

$$G^{*}(\omega )=A_{f}\omega^{1/z}$$ (1)

where ω is the frequency (s^−1^), A_f_ refers to the network strength (Pa s^1/z^) and z to the network connectivity (-) [24]. All measurements were performed in triplicate and average values are presented with standard deviation.

## 3 Results and discussion

### 3.1 Impact of thermal treatment on the shear rheological behavior of dough

**Fig 1:**
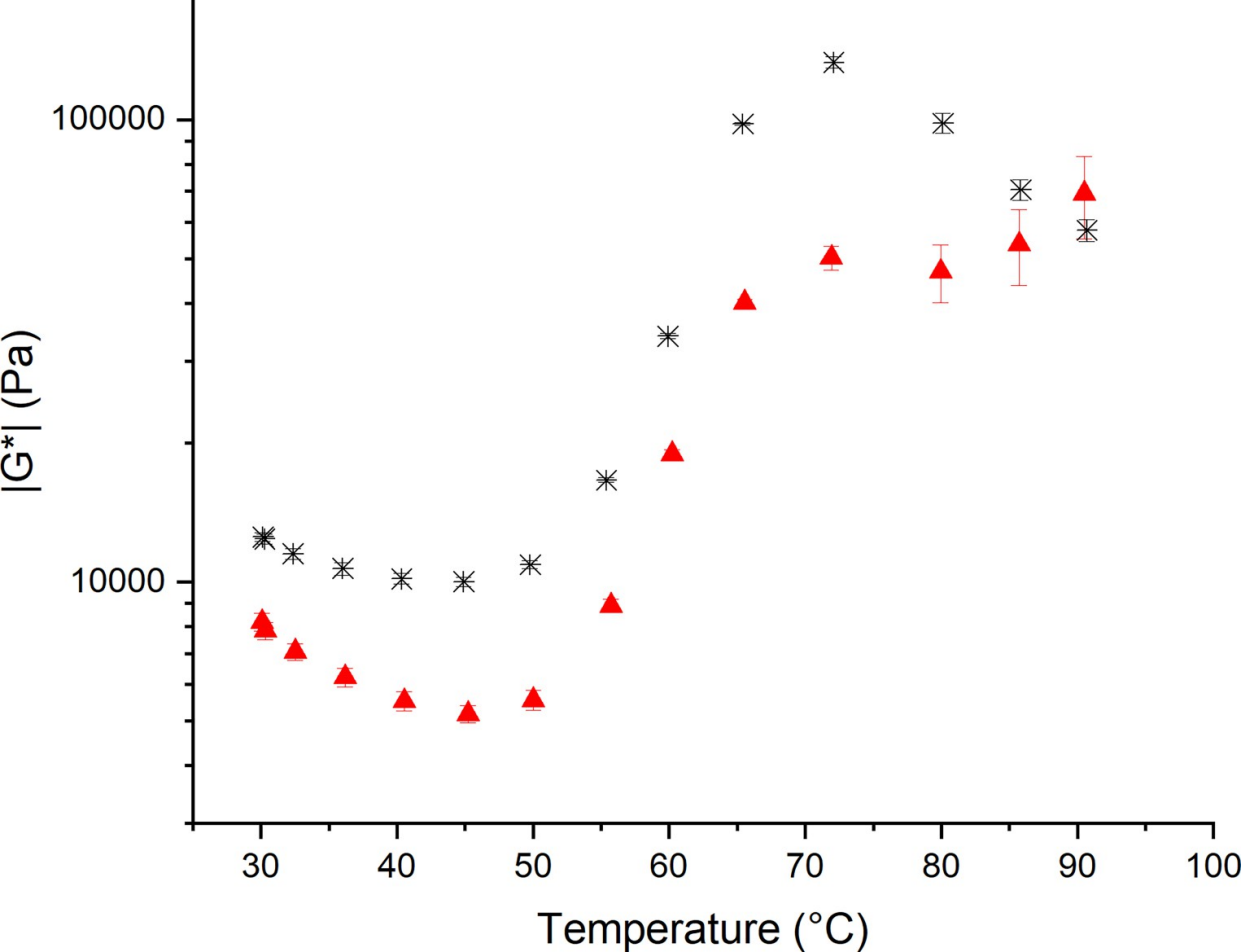
Complex modulus retrieved from shear rheological measurements at *γ* = 0.03% at 1 Hz after 60 min resting/fermenting at 30°C and subsequent heating. (✱) Standard non-yeasted wheat dough and (▲) yeasted wheat dough (1 g fresh yeast/100 g flour). (n ≥ 3, $\bar{X}$ ± STD).

Shear rheology was used to access the functionality of yeasted and non-yeasted wheat flour dough under small deformation. As it can be seen in Fig 1, the initial behavior of both dough samples differs in the absolute value of |G*|, as |G*| of non-yeasted standard wheat dough is higher compared to yeasted wheat dough. This drop in |G*| for yeasted doughs has been observed previously and was related to the entrapped gas cells [18,25–27]. Besides CO_2_, previous work of the authors has shown that secondary yeast metabolites like ethanol or succinic acid only have a minor impact on the rheological behavior of the dough matrix under a small amplitude oscillatory strain [10]. Therefore, the increased gas void fraction, inducing microstructural changes in the protein network (reduced protein strand length and connectivity [10]), of yeasted wheat dough can be regarded as being the decisive factor for the reduction of |G*|. The dependency of |G*| on the gas void fraction can be generally observed in aerated systems, for example in rigid polyurethane foams. For example, Saint-Michel et al. (2006) showed that G‘ increases with an increasing density of these foams [28]. This effect can be explained by a higher material density, causing the interaction of particles to become more probably. This mechanism can be applied to dough matrices with different gas void fractions as well, as dough can be considered as a foamed material [29].

Upon baking, |G*| decreases during the initial heating phase, to a temperature of 46.35 ± 0.04°C or 45.27 ± 0.11°C in case of non-yeasted and yeasted doughs, respectively, followed by a steep increase for both systems. A peak viscosity is reached at 70°C for both dough systems, with |G*| of yeasted doughs being markedly lower than in the non-yeasted system. After this peak viscosity, the behavior differs markedly between the dough systems. The complex modulus of non-yeasted standard wheat dough decreases steadily with increasing temperature, which can mainly be related to the loss of the granular starch structure upon gelatinization. Contrarily, the complex modulus of yeasted dough decreases slightly after the |G*|_max_ and increases again during the final baking phase. This behavior can be explained by the ongoing gas expansion, which is known to cause strain hardening of the extended gluten strands (see Section 3.3). At the end of the baking process, the final behavior resulting from yeasted and non-yeasted dough systems present comparable |G*| values. Therefore, the re-enforcing effect of strain hardening is seen to accomplish for the effects of the higher gas void fraction in yeasted dough. Additional knowledge, underlining the yeast-induced extension of the protein network during the early baking phase in terms of a decreasing protein strand width and increasing average protein strand length, has been reported earlier by accessing the protein network structure by Confocal Laser Scanning Microscopy [21]. The strain hardening effect might further be supported by additional effects, such as altered structures in the dough matrix itself. Here, one reason could be the formation of a denser starch network due to the exclusion of starch granules from the lamella during fermentation and oven rise process. It is therefore hypothesized that the material of the gas cell-surrounding lamellae is mainly composed of gluten strands. Hence, the establishment of starch clusters in the nodes enables a higher extent of starch-starch interactions. It is furthermore hypothesized that starch, being clustered in nodes, might be more accessible to water as the granules are not further embedded in the gluten network. Thus, an accelerated starch gelatinization process could take place in yeasted doughs and the formation of a continuous network of gelatinized starch is more likely to be formed in the nodes [21]. Confirmatory indications were found by accessing the extend of the starch gelatinization process in yeasted and non-yeasted wheat dough systems using Differential Scanning Calorimetry during thermal treatment, where a slightly accelerated starch gelatinization process was observed in yeasted dough systems [21]. Furthermore, the displacement of starch granules changes the strain hardening process, as it is no longer affected by the presence of particles.

**Fig 2:**
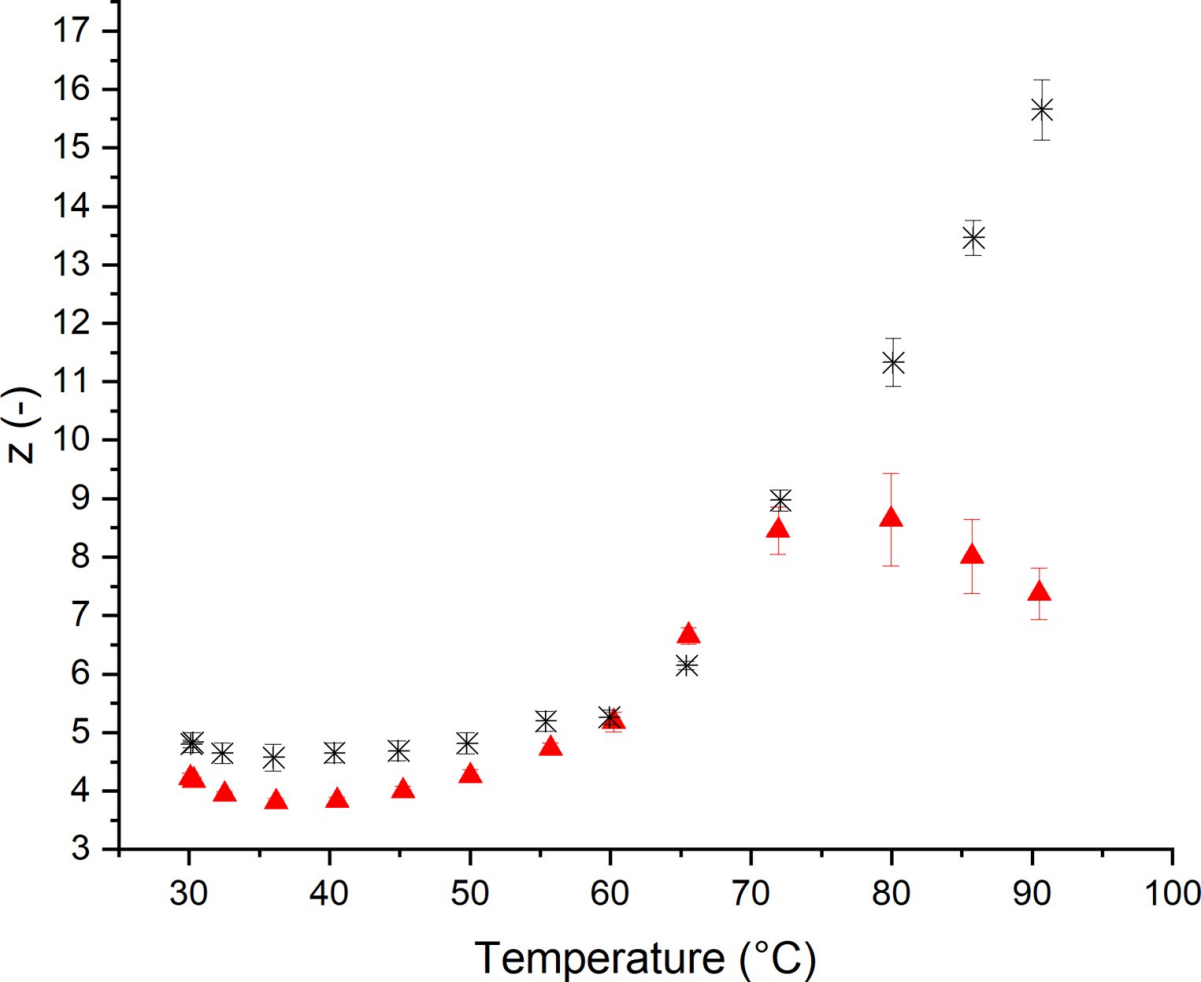
Effect of yeast on the dough structure accessed by shear rheological measurements during the baking process after 60 min resting/fermenting at 30°C. Network connectivity z describing quantity of interactions. (✱) Standard non-yeasted wheat dough and (▲) yeasted wheat dough (1 g fresh yeast/100 g flour) [24,31]. (n ≥ 3, $\bar{X}$ ± STD).

More detailed structural information can be extracted from the applied multiwave approach. The frequency dependent behavior was accessed using a power law fit of the |G*| multiwave data to extract more sophisticated structural information. The obtained values for the network connectivity z are presented in Fig 2. As already assumed previously, the initial non-yeasted standard wheat dough appears to be higher connected, as z of non-yeasted dough is higher than z of yeasted dough. Thus, the yeasted standard wheat dough appears to have a network configuration with less connection points. In this case, the release of CO_2_ is believed to cause a degradation of the network due to a fracture of the dough matrix caused by the expansion of gas cells. This has been observed in the protein microstructures of yeasted dough during the fermentation [10,21]. Upon baking, the z value of non-yeasted standard wheat dough increases steadily due to starch gelatinization and heat induced gluten polymerization [21]. In case of yeasted dough, an abnormal decrease in connectivity was observed for temperatures above 80°C. Above these temperatures, protein polymerization appears to be limited in yeasted dough. As expanding gas cells partially hinder the interaction of protein-protein binding sites, less adjacent proteins can react in gluten polymerization reactions [30]. This can further be supported by the fact that yeasted doughs showed a higher protein extractability, which was formerly related to a limited extent of polymerization in leavened dough systems [21]. Furthermore, the increasing contribution of the thermal expansion of the entrapped gas cells might potentially cause the occurrence of small gluten fragments due to strand rupture. In this case, the initiated degradation counteracts the heat-induced polymerization and leads to a decreasing network connectivity.

### 3.2 Impact of thermal treatment on the extensional rheological behavior of dough

**Fig 3:**
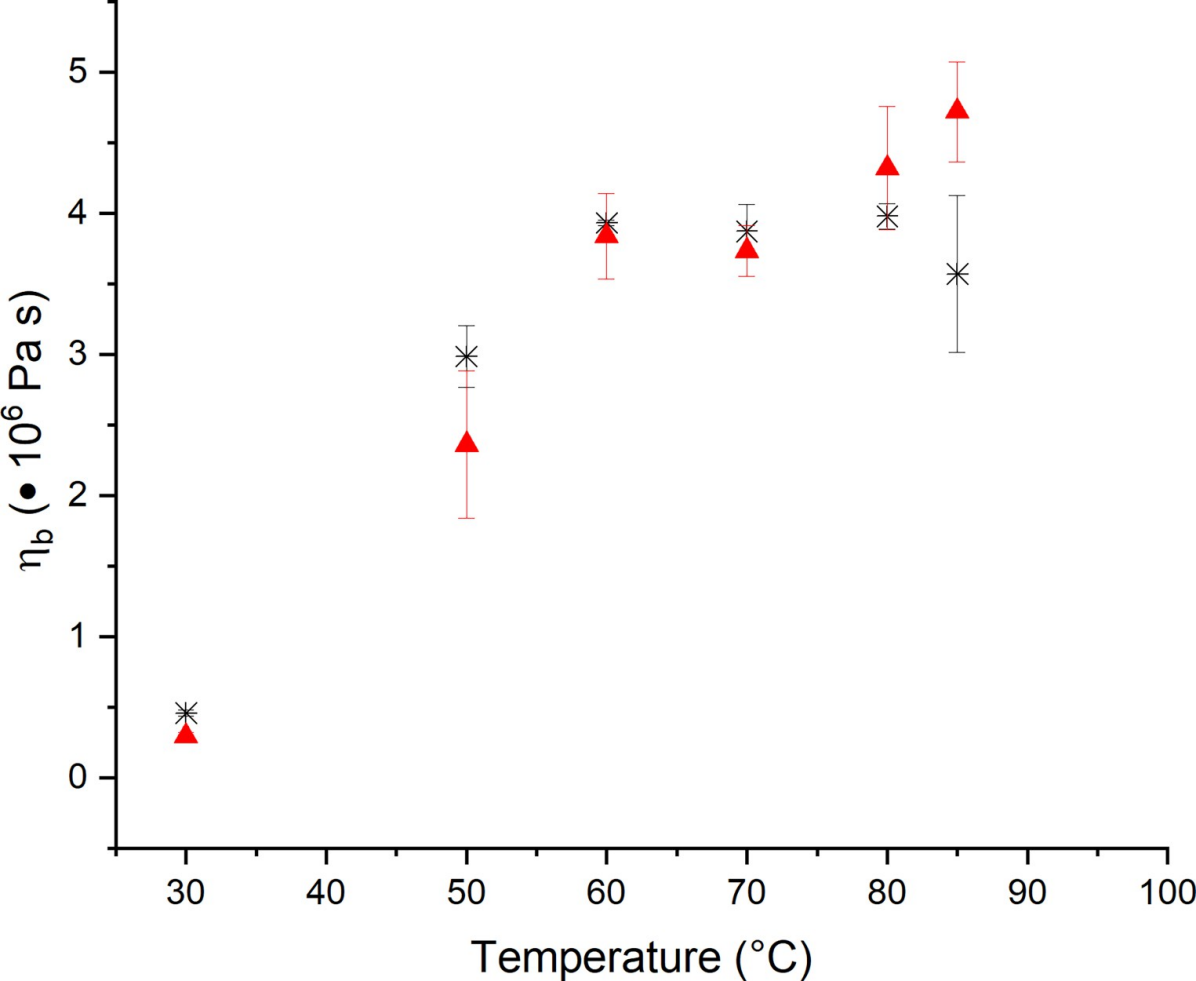
Effect of yeast on the extensional rheological behavior of wheat dough measured at $\dot{\varepsilon_{b}}$ = 0.01 s^-1^ after 60 min resting/fermentation at 30°C and heating to the specified temperature. Viscosities of different dough systems are not quantitative comparable as the mass varied between the different dough systems in order to reach comparable strain—strain rate combinations. (✱) Standard non-yeasted wheat dough and (▲) yeasted wheat dough (1 g fresh yeast/100 g flour). (n ≥ 3, $\bar{X}$ ± STD).

Besides SAOS rheology, large deformation extensional rheology has been used to access the behavior of the dough matrixes. The biaxial viscosity of non-yeasted standard wheat dough and yeasted dough was accessed by LSF. The viscosity values presented do not qualify for an absolute comparison, as the sample masses of non-yeasted and yeasted dough differ. The sample masses used for the measurements were adjusted in order to keep strain-strain rate progression comparable. Therefore, the following section will focus on the dependency of the biaxial viscosity on the temperature rather than focus on a comparison of the absolute values in between dough systems. As can be seen in Fig 3 for an extension rate of 0.01 s^-1^, the viscosity of yeasted dough increases steadily during the whole baking process, whereas non-yeasted dough reaches a plateau viscosity value during the latter baking phase. Thus, the observed behavior differs markedly from the rheological behavior observed for shear rheological testing. While the course of |G*| for SAOS rheology is reminiscent of the course of starch suspensions during heating, the course of η_b_ lacks in the final loss of consistency. As suggested earlier by Turbin-Orger et al. (2016), extensional rheology emphasizes gluten functionality by mainly addressing long-range interactions [32]. It can therefore be concluded that the observed changes of η_b_ are mainly related to changes in the gluten structure and the associated gluten functionality. Overall, the strongest changes in gluten functionality can be observed within 50°C and 70°C, where conformational changes occur due to fewer hydrophobic interactions [30]. For temperatures above 70°C only minor changes appear. Contrarily, yeasted dough systems present a different behavior for η_b_ above 70°C. A further increase of η_b_ until the final baking temperature became evident instead of reaching a plateau value. As discussed above, this might be related to a re-enforcing effect of strain hardening due to the extension of gluten strands in leavened dough or changes in the dough matrix viscosity, such as an altered starch gelatinization behavior. The contribution of strain hardening to the rheological behavior of yeasted dough will be discussed in Section 3.3.

**Fig 4:**
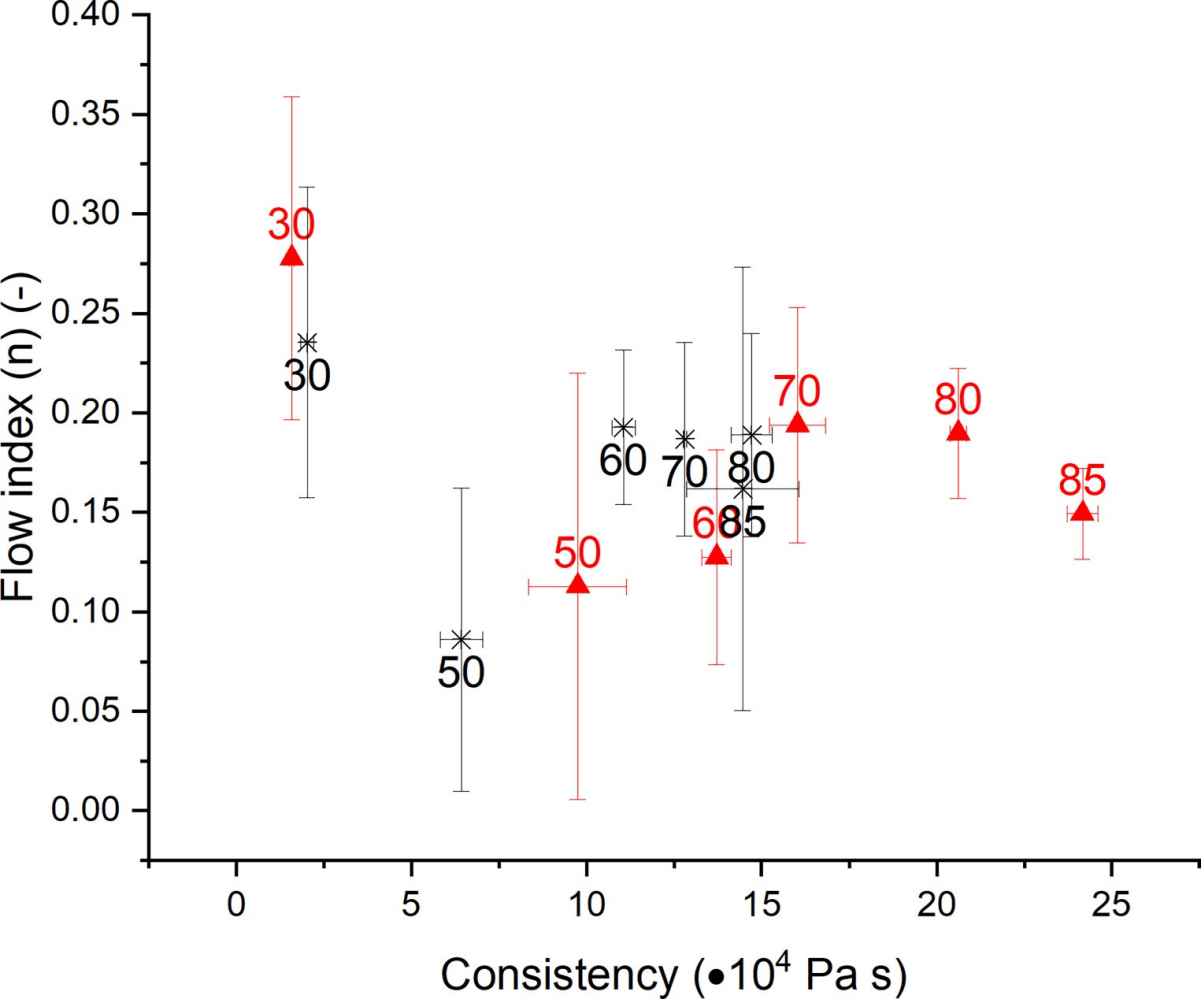
Impact of fermentation and heating on the flow index and consistency for *ε*_*b*_ = 1.00. (✱) Standard non-yeasted wheat dough and (▲) yeasted wheat dough (1 g fresh yeast/100 g flour). The numbers in each value represent the related treatment temperature of the sample. Data of different dough systems is not quantitatively comparable, as the mass varied between the different dough systems in order to reach comparable strain—strain rate combinations. (n = 3, $\bar{X}$ ± STD).

A sophisticated structural interpretation is possible by using the power law model to describe the strain rate dependency of η_b_: $\eta_{b}=K∙{\dot{\varepsilon }}^{\left(n-1\right)}$, where K (Pa s^n^) is the consistency index and n (-) is the flow index [32]. The results in terms of the consistency index K and the flow index n are presented in Fig 4 for non-yeasted and yeasted wheat dough systems for temperatures from 30°C to 85°C. In general, the data points of both dough systems are shown to shift to higher consistency index values with increasing temperature. The consistency index K of yeasted doughs increases steadily from 30°C to 85°C, whereas in non-yeasted doughs, K increases only slightly from 60°C to 80°C and decreases from 80°C to 85°C. The flow index n of standard non-yeasted wheat dough drops initially with a temperature increase from 30°C to 50°C, while K increases for these temperatures. The reduced flow index can be explained using the Arrhenius-like temperature dependency, which states a higher mobility of the polymers with increasing temperature. These initial changes are followed by a steady increase of n with K till final values for n (final flow index reached at 60°C) and K (final value reached at 80°C) are reached. Therefore, the flow index seems to be limited by the onset of the starch gelatinization process, which was formerly shown to occur within 50°C and 70°C [21]. Regarding the consistency index, a leveling off only after the setting in of the protein polymerization process can be observed. The onset of the polymerization process has been located earlier by protein extractability measurements and was found to set in at temperatures above 65°C or 70°C [21,30].

Contrarily, the behavior of the yeasted wheat dough has been shown to be markedly affected by the presence of growing gas cells. After the likewise appearing initial drop, n shows the highest flow indices at 70 and 80°C, followed by a steady decrease of the flow index. This can be explained by an increasing crosslinking, which is then limited by an increasing network degradation due to growing gas cells and the accompanied overextension of the lamella. Contrarily, K of yeasted wheat doughs shows a steady increase, even during the final baking phase. As this behavior cannot be observed in non-yeasted doughs, this behavior is therefore likely to be explained by the strain hardening phenomena triggered by the extending gas cells. Therefore, the presented results suggest different mechanisms behind the solidification behavior of non-yeasted and yeasted dough matrices. While pure polymerization appears to dominate the solidification process in non-yeasted dough systems, yeasted dough undergoes limited polymerization, but more pronounced strain hardening due to the expansion of gas cells. This hypothesis can be underlined by the results of SDS-supported protein extraction of heat-treated wheat dough systems as presented by Alpers et al. (2022). The study revealed a lower extend of protein polymerization in yeasted wheat dough for temperatures above 65°C, compared to non-yeasted wheat dough. This was explained by a higher gas void fraction in yeasted dough, which limits the connectivity of the protein strands and, therefore, the amount of proteins in reactive distance for polymerization [21,30]. On the other hand, as discussed above, microstructural data on the protein network reveals a decreasing protein strand width, indicating gas cell growth during the initial baking process [21]. Therefore, initialization of strain hardening behavior is likely to be more pronounced in yeasted wheat dough, counteracting the functional effects of a lower extend of protein polymerization.

### 3.3 Impact of thermal treatment on the strain hardening behavior of dough

**Fig 5:**
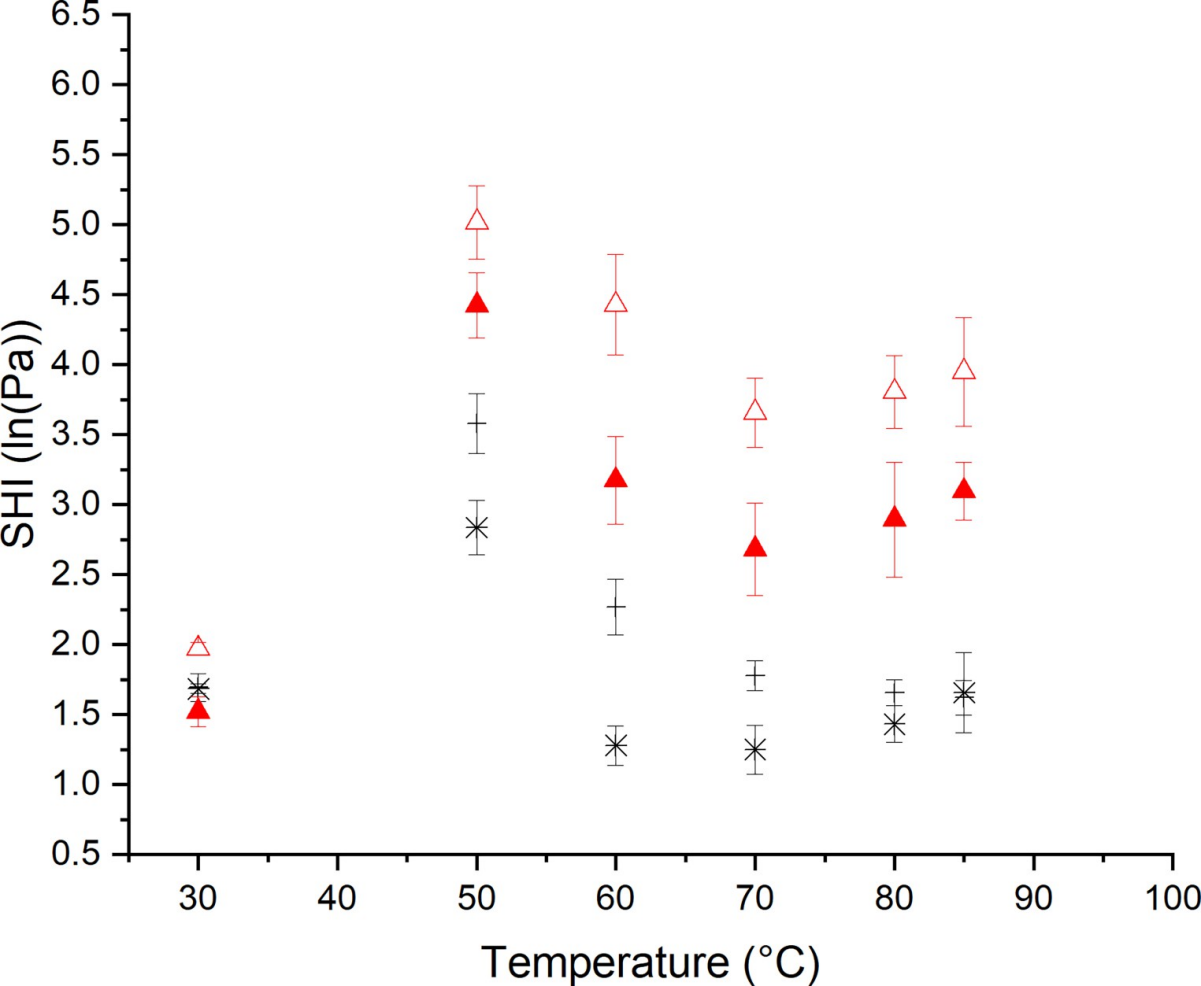
Strain hardening behavior of non-yeasted and yeasted dough after 60 min fermentation and subsequent heating to the specified temperatures. (✱) Standard non-yeasted wheat dough, $\dot{\varepsilon_{b}}$ = 0.01 s^-1^, (―) standard non-yeasted wheat dough, $\dot{\varepsilon_{b}}$ = 1.00 s^-1^, (▲) yeasted wheat dough (1 g fresh yeast/100 g flour), $\dot{\varepsilon_{b}}$ = 0.01 s^-1^ and (△) yeasted wheat dough (1 g fresh yeast/100 g flour), $\dot{\varepsilon_{b}}$ = 1.00 s^-1^. (n = 3, $\bar{X}$ ± STD).

To further elucidate the ability of both matrices for stabilization by strain hardening during the baking process, this phenomenon was quantified. The dependency of the strain hardening functionality on the extent of the thermal treatment is presented in Fig 5. Up to a temperature of 50°C, a strong increase of the SHI for both dough systems can be observed. This can mainly be related to the thermally induced changes in protein conformation and interactions. The decreasing strength of hydrogen bonds limits the ability of the gluten strands to interact via short-range interactions. Due to this, the slippage of proteins along each other without intermediate stabilization is more likely to cause strain hardening. Furthermore, the initiation of conformational changes causes the aggregation of proteins and changes the ability of extension of loop regions upon elongation. This might explain the early triggering of strain hardening due to the reaching of the extensibility limit. For temperatures below 50°C, the pasting process of starch is also known to reduce the overall mobility of the system and therefore causes a decreasing plasticization of the polymers [33]. With progressive thermal treatment, a decreasing strain hardening index can be observed within 50°C and 70°C. Here, the gelatinization of starch decreases the rigidity of starch granules and leads to the development of a continuous starch-gluten network. Additional binding sites might therefore support the extension processes and enable intermediate stabilization. Additionally, dissociating starch granules are hypothesized to cause less friction on the extending gluten strands and therefore induce less pronounced strain hardening, which can explain the decreasing SHI as well. During the final baking phase, the SH index is shown to increase again, whereby the yeasted dough system presents a more pronounced SHI increase. At the molecular level, the polymerization process of gluten proteins has been reported to initiate at temperatures above 70°C [30]. Increasing molecular interactions [30] could therefore decrease the amount of extendable loop regions. The extension process is therefore hypothesized to be limited due to the limited expendability of the protein strands. Thus, the strain hardening process is triggered already at a lower overall extension and the measured SHI is increasing.

Additional information can be obtained by comparing the strain hardening behavior of the dough systems at different strain rates. Furthermore, the strain rate dependent behavior can be used to predict the behavior of the dough systems under different deformations along the bread making process. For this reason, the current study uses two strain rates to predict the behavior of the dough systems at slow and fast extension processes. At an extension rate of $\dot{\varepsilon }$ = 0.01 s^-1^, the SHI of both dough systems was found to be lower than at the fast extension rate of $\dot{\varepsilon }$ = 1.00 s^-1^. This behavior has previously been observed by the authors and was explained by the enabling of rearrangement, slippage and intermediate stabilization along protein strands at slow extension rates [10]. These processes were hypothesized to lower the strain hardening index for dough systems at low extension rates. In case of high extension rates, gluten strands might remain entangled due to the fast extension process. In this case, no free slippage and missing stabilization by intermediate polymer–filler-interactions are hypothesized to contribute to an intensified strain hardening behavior. Additionally, friction occurring within starch granules or starch granules and protein strands might raise the strain hardening behavior. The observation that the SHI is particularly high around 50°C, where starch is still in its granular structure, enforces the hypothesis of friction contributing to strain hardening behavior. Considering the SHI at fast extension rates, a more pronounced strain hardening behavior becomes evident for yeasted dough systems. The pre-extended protein strands of the yeasted dough system seem to cause an earlier onset of strain hardening, which leads to an overall increase of the SHI for yeasted dough systems. This proves the previously considered explanation of a higher strain hardening character of yeasted dough systems, which was used to explain the observed differences in shear and elongational rheological behavior of both dough systems, especially at higher temperatures.

### 3.4 Oven rise behavior and macroscopic quantification of extension processes

**Fig 6:**
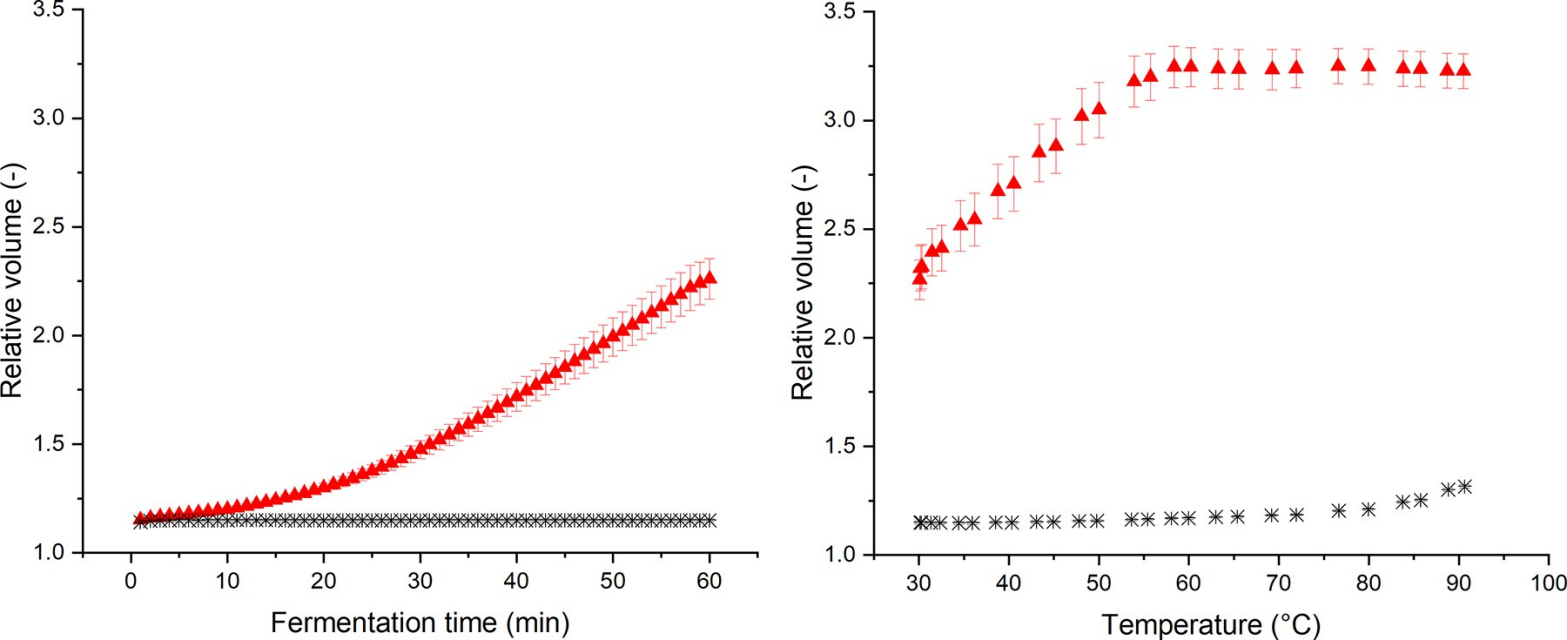
Relative volume of yeasted dough and non-yeasted standard wheat dough during 60 min fermentation at 30°C and subsequent heating to 90°C. a) Time dependency of the relative volume during the fermentation process. b) Temperature dependency of the relative volume during the heating process. (✱) Standard non-yeasted wheat dough and (▲) yeasted wheat dough (1 g fresh yeast/100 g flour). (n ≥ 3, $\bar{X}$ ± STD).

Using the knowledge on strain-dependent dough functionality in different matrices gained during the baking process, the oven rise behavior of both matrices during the baking process will be discussed in the following section. For this reason, the course of the relative dough volume for yeasted and non-yeasted wheat dough during fermentation and baking are presented in Fig 6. It becomes evident that the yeast dough system is subjected to a significant volume expansion during the fermentation process (Fig 6A). The course of the increase of the relative volume is similar to the course reported by Chevallier et al. (2012) [34]. As stated by those authors, the growth can be divided into different phases. During an initial lag phase, the incorporated yeast cells produce CO_2_, which migrates in the liquid dough matrix and remains solubilized. This phase is followed by an initial growth phase. After the solubility limit of the liquid dough phase has been reached, CO_2_ starts to dissolute and dissipates into the entrapped gas cells. The increasing gas fraction causes the expansion of the gas cells. This leads to the establishment of a linear growth rate, with which the expansion process continues throughout the whole 60 min of fermentation [34]. During this period, no stationary phase is reached and hence, the gas holding limit of the dough systems is not exceeded. Consequently, the produced CO_2_ can still be entrapped and stabilized within the dough matrix and does not escape yet. Contrarily, the non-yeasted dough is not subjected to any considerable volume expansion processes. The relative volume of the system is rather inert during the resting phase, which indicates no initial inoculation and no microbial contamination of the flour with relevance for gas formation.

After the fermentation phase, the yeasted dough starts with a higher initial relative volume into the baking process. During the baking phase, further expansion processes can be observed (Fig 6B). This expansion is referred to as oven rise, which is defined as the volume expansion of dough during the thermally induced dough to crumb transition. In case of yeasted dough, a steep initial oven rise behavior can be observed up to 50°C. The maximum relative volume is then reached at 60°C and retained until the end of the baking process. The initial expansion can be explained by the CO_2_ production of yeast cells till the death of yeast cells sets in at ~ 50°C [35–37]. During this initial baking phase, an expansion rate of 0.104 min^-1^ was determined by a linear model (relative volume–baking time), which is markedly higher compared to the gas production rate during the fermentation phase. During this phase, the expansion rate was quantified to 0.027 min ^-1^. The differences can be explained by the well-known thermal dependency of yeast activity. Due to this dependency, a higher gas fermentation rate can be observed at elevated temperatures up to a temperature of 50°C, where yeast cells are subjected to thermal damage. Additionally, thermally driven expansion processes promote the volumetric expansion of the matrix while baking. The yeast-driven oven rise is followed by another short expansion phase till 60°C. The appearing expansion can now be clearly related to further expansion of the already incorporated gas phase, evaporating water and ethanol, dissolution of CO_2_ and thermal expansion of the liquid dough phase within 50°C and 60°C. Beyond 60°C, no further expansion can be observed. As previously observed in the quantification of the shear and elongational rheological behavior, yeasted dough has a limited network connectivity and enhanced strain hardening behavior. This could lead to a limited gas holding capacity and repressed final oven rise as the viscosity increases in a more pronounced way due to strain hardening.

Contrarily, oven rise in standard wheat dough was found to be comparably low due to the limited amount of gas entrapped in the dough matrix. A significantly retarded oven rise can be observed (starting at 70°C). The end of the expansion process was not recorded within the conducted experimental setup. The rheological characterization revealed a softening of the dough matrix for temperatures above 70°C, quantified in terms of a decreasing |G*|. This might promote an expansion during the latter baking phase. Additionally, no decrease in network branching was quantified (n and z remained constant or increased). Therefore, there is no indication for a limited gas holding capacity in case of standard non-yeasted wheat dough which could limit further oven rise as it was shown for the yeasted system, which suffered remarkable damage on a microstructural level. The oven rise of the standard wheat dough system, which is induced by evaporation processes, results in an expansion rate of 0.035 min^-1^. As the expansion process proceeds slower than in yeasted dough, less strain hardening is to be expected due to the fact that slow extension processes cause less strain hardening compared to fast extension processes (see Section 3.3). The lower resistance to extension of the dough matrix might therefore contribute to the occurrence of oven rise even at high temperatures, even though the lower relative volume has to be taken into account, favoring oven rise during the final baking phase as well.

## 4 Strain-dependent functionality of the dough’s polymers during the baking process: A structural model

The major focus of this work was the identification of different functional structures within the dough matrix, which respond to certain types and magnitudes of exerted strain. As the bread making process involves deformations of various types and strengths, the response of the dough matrix composing polymers to these stresses is important so the behavior of the system during the production process can be understood. Especially during the baking step, where conformational transition processes of the dough’s composing polymers are initiated, the changing functionality of each component is of great interest. Therefore, SAOS rheology and large deformation extensional rheology (LSF) were applied to a non-yeasted standard wheat dough system and a yeasted wheat dough system during thermal treatment.

Our results show that the response to the application of small deformations is markedly different to the response to large deformations. While small strains mainly resulted in the response of filler-filler interactions, large deformations gave rise to more pronounced gluten functionality. This agrees with the observations of Turbin-Orger et al. (2016) [32]. With this knowledge, the contradicting behavior and structural evolvement of both systems ((i) highly connected standard wheat dough and (ii) pre-extended and structurally degraded yeasted wheat dough under conventional shear rheology can be explained: The standard wheat dough system (i) mainly depicts starch functionality upon thermal treatment. Starch pasting and gelatinization initiated a first raise in viscosity around 45°C and lead to a maximum peak viscosity around 70°C, where starch gelatinization was formerly shown to be fairly concluded. Continued thermal treatment led to a viscosity drop due to the increased mobility of starch molecules. Structural information obtained by the application of the multiwave technique revealed an increasing structural connectivity during this final heating phase, which may be attributed to increased interactions within starch molecules in the new-formed continuous starch phase. Besides this, the heat-induced gluten polymerization contributes to the increased connectivity. The increasing connectivity of the standard wheat dough system during the final baking phase also becomes evident when looking at the behavior of standard wheat dough under large elongational deformation: Even though starch functionality would cause a viscosity drop in the final baking phase, this behavior is strongly reduced when considering η_b_. By evaluating the SHI, even a hardening above 80°C becomes obvious due to heat-induced gluten polymerization.

In yeasted dough systems (ii), a higher share of gluten functionality was shown to be initiated as the leavening of the system caused additional deformation. These internal forces caused a pre-extension of the system (as quantified on a microstructural level by [21]) and therefore favored the occurrence of large deformation behavior. Therefore, the behavior of yeasted doughs under a small deformation showed a comparable course to the behavior observed under a large extensional deformation. The usage of small as well as large deformation techniques further provided good insights into the effect of yeast fermentation on the dough matrix structure. In general, yeasted wheat dough appeared to be less well connected, which gave rise to a reduced matrix strength (lower complex modulus) This observation can be supported by comparative data on the extend of protein polymerization in yeasted and non-yeasted wheat dough as reported by Alpers et al. (2022), which indicates a higher level of non-crosslinked, low molecular weight gluten in yeasted wheat dough during the final baking process. The fitting of the observed rheological behavior under both, small and large deformation, by structural models revealed a strong impact of the expanding gas cells on the course of the connectivity of the dough matrix during the baking procedure. Starting with an already less connected network, the expanding gas cells were further shown to impact starch gelatinization and heat-induced gluten polymerization. In case of gluten functionality (quantified by large deformation rheology), the polymerization (especially above 70°C) was shown to be limited, as the mobility of the system seemed to be rather reduced by an increasing consistency (e.g. strain hardening) than by an increase in connectivity. The opposite was observed in starch functionality under small deformations: Upon the expansion of gas cells, starch granules are hypothesized to be excluded from the lamella and clustered in the connecting nodes. The clustering of starch in nodes gave rise to an earlier and more extensive starch gelatinization process in yeasted doughs (slight shift in the inflection point to a lower temperature). Further, the aggregated starch polymers were shown to be more likely to form a continuous network within the nodes when the granular structure breaks up in succession of the gelatinization process as the strength of the network under small deformations increased during the final baking step, while the connectivity itself was shown to decrease.

The importance of starch and gluten functionality for the oven rise process became evident when the gained knowledge on structural changes is related to the functionality of the systems. In case of yeasted dough, a strong oven rise behavior was observed during the initial baking phase. This can mainly be related to the thermal activation of yeast, which causes the more rapid production and dissolution of CO_2_. As gluten functionality is strongly preserved (high connectivity (z and n), strong strain hardening behavior), the released gas can be stabilized in the dough matrix. During the latter baking process (above 60°C), no further expansion can be observed. Based on the elucidated structural changes within the system, structural degradation dominates in this point. Here, decreasing connectivity indicates rupture of the network, together with the presence of smaller, highly mobile gluten fragments [21]. Consequently, the SHI strongly decreases and any further gas released (e.g. by dissolution, evaporation or expansion processes) can no longer be stabilized by the dough matrix. Besides this, the expansion of yeasted wheat dough can be considered a rather fast expansion process as a high amount of gas stabilized in the system expands and considerable amounts of dissolving CO_2_ cause a fast increase of the gas void fraction. The occurring fast expansion rates therefore initiate a higher resistance (SHI) of the system towards further expansion, which might limit the extensibility during the final baking phase additionally. Contrarily, in non-yeasted dough, expansion processes are rather slow, as the gas cell expansion is mainly based on evaporation and thermal expansion processes. During slow expansion processes, intermediate stabilization mechanisms were previously shown to be active and able to stabilize the matrix expansion [10]. Furthermore, starch functionality should be considered different during slow expansion processes, as the occurring friction is low and rather a stabilizing effect was hypothesized due to the possibility of intermediate stabilization within starch and gluten upon the reformation of short range interactions upon expansion. As starch functionality was shown to dominate the small deformation response of the system, a stabilizing effect can be attributed to starch during the expansion process of non-yeasted dough during the final baking phase. The low viscosity measured under small deformation processes during the final baking phase and the limited strain hardening functionality for slow extension processes are both attributed to the preserved oven rise functionality above temperatures of 80°C in non-yeasted dough. Hence, it is necessary to quantify the rheological behavior of dough systems according to the type and strength occurring during the production process to extract reliable data.

## 5 Conclusion

By elaborating inline fermentation and baking techniques, allowing the assessment of both—yeasted and non-yeasted dough systems -, it was possible to determine the effect of structural changes of the main dough’s polymers on their changing functionality in the dough matrix during the baking step. In this framework, yeast was used as a tool to implement structural changes in the dough matrix without changing the functionality of the dough’s polymers itself. Subjecting these systems to different types and magnitudes of strain upon thermal treatment, a structural model was proposed for the dough’s polymers during baking. The contribution of the functionality of the polymers to accomplish an oven rise has been elucidated by differentiating the functionality of the polymeric system in dependency of the experienced thermal treatment and deformation occurring within the dough matrix. Overall, SAOS multiwave rheology was shown to be a powerful tool to analyze starch functionality in non-yeasted systems. Contrarily, large deformation techniques, initiated either by external or internal forces, have been shown to be more impacted by gluten functionality due to the enhanced strain hardening behavior, caused by gluten extension or the stress memory of gluten.
